# Effect of dexmedetomidine on somatosensory- and motor-evoked potentials in patients receiving craniotomy under propofol-sevoflurane combined anesthesia

**DOI:** 10.3389/fsurg.2024.1386049

**Published:** 2024-07-09

**Authors:** Xue Yang, Xinyi Zhang, Puxuan Lin, Zeheng Liu, Shuhang Deng, Shanwen Liang, Xinyi Zhu, Qianqian Qiao, Qianxue Chen

**Affiliations:** ^1^ Department of Neurosurgery, Renmin Hospital of Wuhan University, Wuhan, China; ^2^ Department of Anesthesiology, Renmin Hospital of Wuhan University, Wuhan, China

**Keywords:** dexmedetomidine, transcranial motor-evoked potentials, intraoperative neurophysiological monitoring, neurosurgery, prognosis

## Abstract

**Introduction:**

Dexmedetomidine is often used as an adjunct to total intravenous anesthesia (TIVA) for procedures requiring intraoperative neurophysiologic monitoring (IONM). However, it has been reported that dexmedetomidine might mask the warning of a neurological deficit on intraoperative monitoring.

**Methods:**

We reviewed the intraoperative neurophysiological monitoring data of 47 patients who underwent surgery and IONM from March 2019 to March 2021 at the Department of Neurosurgery, Renmin Hospital of Wuhan University. Pre- and postoperative motor function scores were recorded and analyzed. Dexmedetomidine was administered intravenously at 0.5 μg/kg/h 40 min after anesthesia and discontinued after 1 h in the dexmedetomidine group.

**Results:**

We found that the amplitude of transcranial motor-evoked potentials (Tce-MEPs) was significantly lower in the dexmedetomidine group than in the negative control group (*P* < 0.0001). There was no statistically significant difference in the somatosensory-evoked potentials (SSEPs) amplitude or the Tce-MEPs or SSEPs latency. There was no significant decrease in postoperative motor function in the dexmedetomidine group compared with the preoperative group, suggesting that there is no evidence that dexmedetomidine affects patient prognosis. In addition, we noticed a synchronized bilateral decrease in the Tce-MEPs amplitude in the dexmedetomidine group and a mostly unilateral decrease on the side of the brain injury in the positive control group (*P* = 0.001).

**Discussion:**

Although dexmedetomidine does not affect the prognosis of patients undergoing craniotomy, the potential risks and benefits of applying it as an adjunctive medication during craniotomy should be carefully evaluated. When dexmedetomidine is administered, Tce-MEPs should be monitored. When a decrease in the Tce-MEPs amplitude is detected, the cause of the decrease in the MEPs amplitude can be indirectly determined by whether the decrease is bilateral.

## Introduction

Intraoperative neurophysiological monitoring (IONM) is often used in neurosurgery to assess the functional integrity of target neural structures (1, 2). The most commonly used electrophysiological methods include somatosensory-evoked potentials (SSEPs), motor-evoked potentials (MEPs) or transcranial motor-evoked potentials (Tce-MEPs), brainstem auditory-evoked potentials (BAEPs), electroencephalography (EEG), and electromyography (EMG). Among them, somatosensory-evoked potentials (SSEPs) are electrical signals recorded from the scalp or spine following stimulation to peripheral nerves. They are time-locked responses, representing the function of the ascending sensory pathways (3). In central nervous system surgery, especially during lumbar spine surgery, SSEPs are highly specific and moderately sensitive in predicting new postoperative neurological deficits (4, 5). Transcranial motor-evoked potentials (Tce-MEPs) are widely used in brain motor area tumor surgery and are the most commonly used method for monitoring motor-evoked potentials (MEPs). By monitoring Tce-MEPs, neurosurgeons can assess the integrity of the patient's motor conduction pathways in real time during craniotomy, thereby maximizing the removal of the lesion and preserving motor function, which helps to reduce the incidence of postoperative complications such as paralysis (6–11). Combined monitoring of Tce-MEPs and SSEPs to reduce postoperative new motor deficits and sensory deficits is the method of choice for central nervous system (CNS) surgery.

It is a challenging task to achieve good anesthesia while obtaining reliable EPs during craniotomy. To date, the most common inhalation anesthetics inhibit the amplitude of evoked potentials (EPs), and this inhibition is both dose-effective and dose-specific and becomes more profound with the deepening of anesthesia (12–14). At clinically relevant doses, propofol is considered to have a lower inhibitory effect on the amplitude of EPs than inhaled anesthetics. To reduce the interference of anesthetics on the amplitude of EPs, most researchers currently advocate for the use of propofol-based total intravenous anesthesia (TIVA) during IONM (15, 16). Small doses of sevoflurane are also used to maintain a minimum alveolar concentration (MAC) below 0.5 due to its physical stability, rapid onset of action, and low circulatory depression (17, 18), and it is often applied in combination with propofol in cranial surgery. However, there seems to be a limit to propofol plasma concentrations above which Tce-MEPs monitoring is compromised (19). To avoid this situation and reduce the dosage of propofol, a number of adjuvant drugs have been used in TIVA.

Dexmedetomidine, a highly selective *α*-2 adrenergic agonist, has physiological effects, including analgesia, sedation, and neuroprotection, and it facilitates smooth recovery of patients from general anesthesia and reduces the need for opioids after surgical procedures. Dexmedetomidine has been proven to be able to reduce the need for anesthesia with inhaled anesthetics and propofol, leading to a reduction in the dose of propofol by approximately 50% (20). Currently, dexmedetomidine is increasingly used as an adjunct to total intravenous anesthesia (TIVA) (13, 21–25). However, it has been reported that the addition of dexmedetomidine as an adjunct to TIVA in central nervous system surgery has an impact on the accuracy of intraoperative neurophysiological monitoring and that dexmedetomidine might decrease the amplitude of motor-evoked potentials (26–28). Therefore, the present study collected the data of 47 patients, with the aim of analyzing the characteristics of the suppressive effect of dexmedetomidine on Tce-MEPs and exploring the causes of the effect. We hope to provide a basis and guidance for clinical treatment through this study.

## Methods

### Patient selection and demographic data

**Figure 1:**
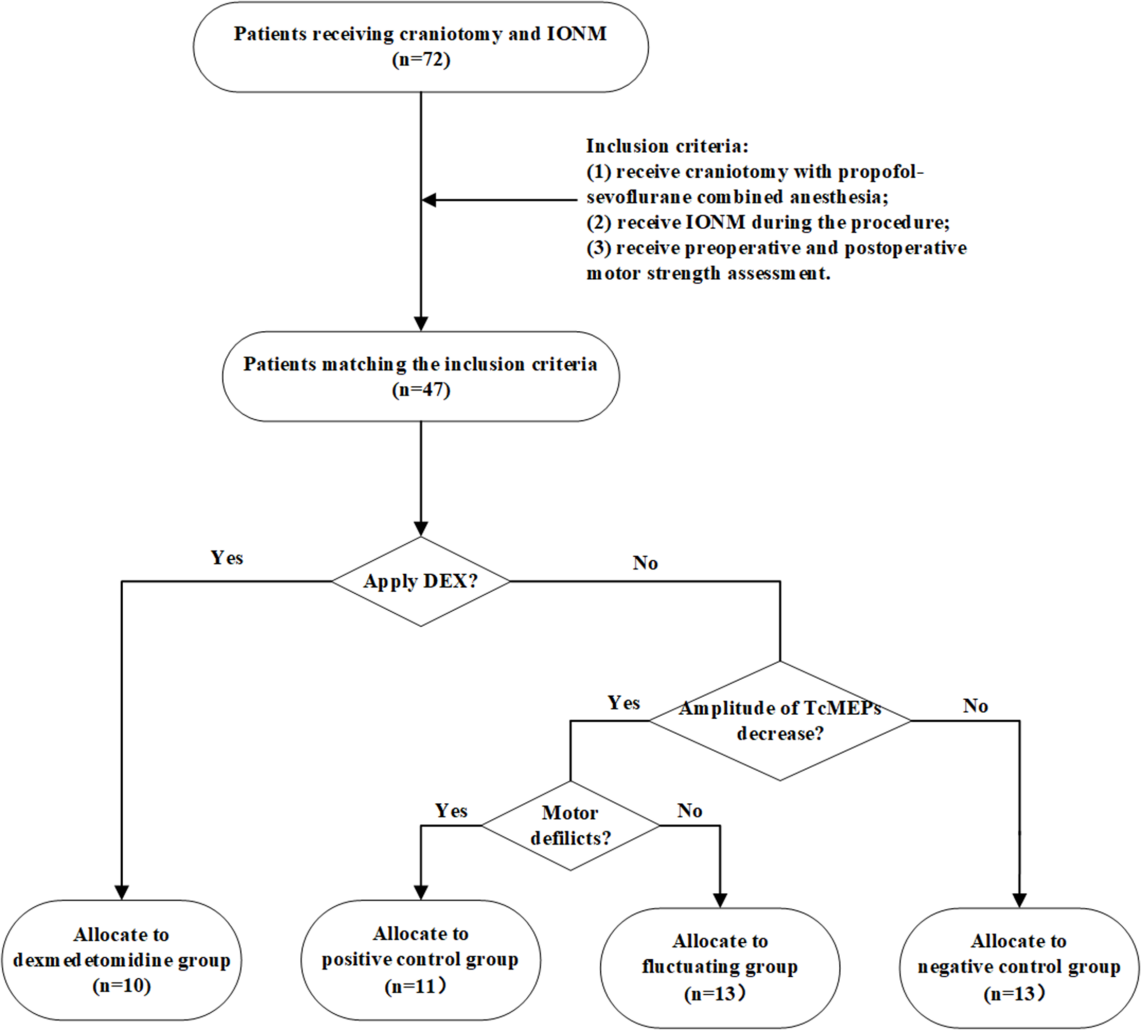
Flowchart of the patient selection and grouping. We screened 47 patients who met the inclusion criteria from 87 patients in total and categorized them into four groups according to whether dexmedetomidine was applied intraoperatively, whether there was a decrease in the amplitude of Tce-MEPs, and whether there were postoperative motor deficits. DEX means dexmedetomidine.

Seventy-two patients who underwent craniotomy and IONM between March 2019 and March 2021 at the Department of Neurosurgery, Renmin Hospital of Wuhan University, were enrolled in this study. Figure 1 shows the flowchart of the patient selection and grouping. The exclusion criteria were as follows: (1) poor quality baseline waveforms; (2) patients who were unable to use the preset propofol-sevoflurane combined anesthesia regimen due to medical conditions; (3) patients with a history of alcohol or drug abuse; (4) patients who experienced an irreversible decrease in the Tce-MEPs amplitude during surgery; and (5) patients whose postoperative data were not collected due to loss to follow-up. A total of 47 patients who met the inclusion criteria were divided into the following four groups: negative control group, dexmedetomidine group, positive control group, and fluctuating group.

The present study aimed to more clearly explore the effect of dexmedetomidine on the reliability of Tce-MEPs by establishing these four groups. In the negative control group, propofol-sevoflurane combined anesthesia without dexmedetomidine was applied and there were no postoperative motor deficits. In the positive control group, the Tce-MEPs amplitude decreased on the injured side (>50%) and the corresponding postoperative motor deficits were caused by intraoperative injury to the motor function area of the brain. The negative control group and positive control group separately demonstrated the effects of the conventional TIVA regimen and intraoperative brain injury on IONM data and set a baseline. Moreover, a fluctuating group was also established to observe the influence of a one-time decrease in the Tce-MEPs amplitude on the recovery of postoperative motor function. In the fluctuating group, the decrease in the Tce-MEPs amplitude reached the warning value (50%), but due to timely intraoperative remediation, the Tce-MEPs amplitude rebounded and eventually, there was no change in postoperative motor function.

This study was performed with the approval of the Ethics Committee of Renmin Hospital of Wuhan University, under reference number WDRY2021-KS003.

### Motor strength assessment

Motor strength was assessed by the Medical Research Council (MRC) scale, which is a 0 to 5 scale routinely used to assess patient motor function, and was recorded preoperatively and postoperatively from the electronic medical records. The muscle group with the lowest score was noted for each limb. Motor deficits were defined by a decrease in the respective extremity motor function score compared to the preoperative score at each postoperative time point.

### Study protocol

**Figure 2:**
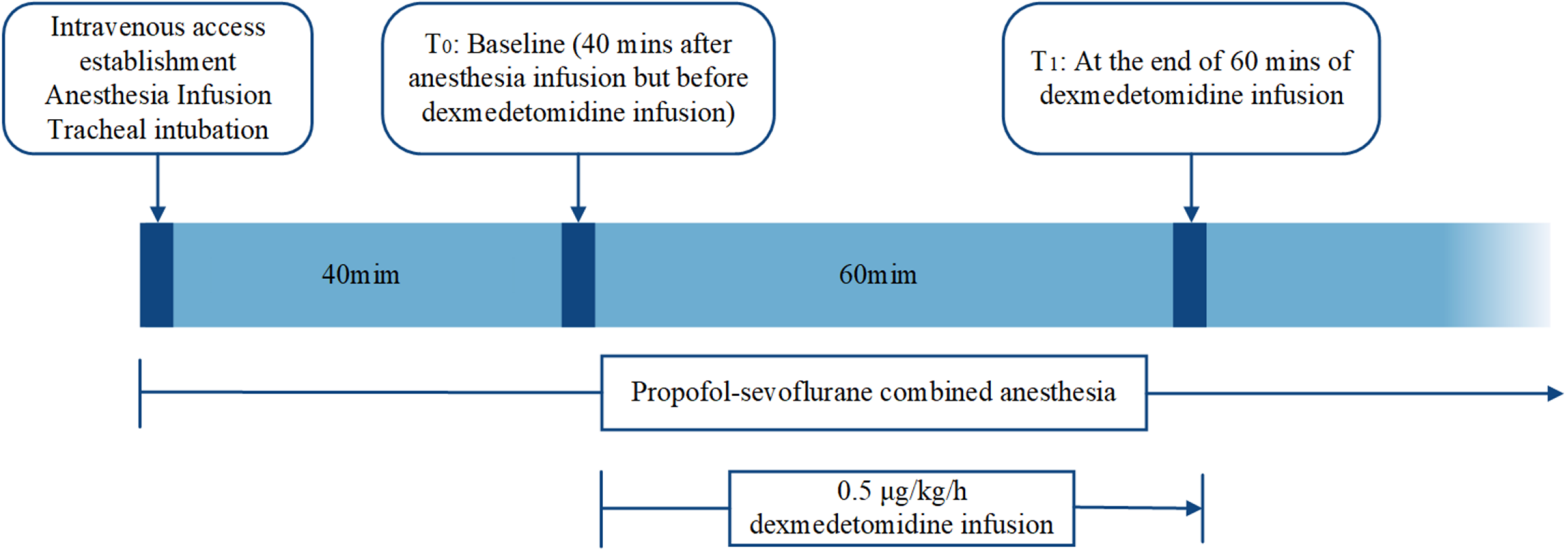
Flow chart of the study protocol. Dexmedetomidine was infused intravenously at 0.5 μg/kg/h 40 min after anesthesia and discontinued after 1 h in the dexmedetomidine group. Propofol-sevoflurane combined anesthesia was used in all the groups. Intraoperative vital signs were collected and recorded by the study coordinator at T0 and T1.

All patients received propofol-sevoflurane combined anesthesia. After establishing intravenous access, propofol and remifentanil at plasma concentrations of 2∼4 mg/ml and 3∼8 g/ml, respectively, were infused using a target-controlled infusion pump for the induction and maintenance of anesthesia. After induction, tracheal intubation was facilitated with cisatracurium (0.2 mg/kg) and sufentanil (0.3 μg/kg). Sevoflurane was used to maintain the MAC at 0.5. To minimize the interference of cisatracurium and sufentanil in the monitoring of the effects of dexmedetomidine, dexmedetomidine was added after cisatracurium and sufentanil were metabolized. In addition, since this was a retrospective clinical study and the effect of dexmedetomidine on IONM parameters is still controversial, dexmedetomidine was not applied intraoperatively for an extended period. Overall, according to the judgment of our anesthesiologists, dexmedetomidine was administered intravenously at 0.5 μg/kg/h 40 min after anesthesia and discontinued after 1 h in the dexmedetomidine group, whereas dexmedetomidine was not used in the other groups. Neostigmine and pyrrolose were not used to reverse intraoperative neuromuscular blockade. Intraoperative monitoring included continuous ECG, pulse oximetry, blood pressure, and core body temperature measurements, which were assessed by esophageal probes. Intraoperative vasoactive drugs were used to tightly control and maintain the mean arterial pressure within 20% of the preoperative measurements. The core body temperature was maintained at 35.5°C to 37°C, and end-expiratory carbon dioxide was maintained in the range of 35∼45 mm Hg (Figure 2).

### Neurophysiological monitoring

**Figure 3:**
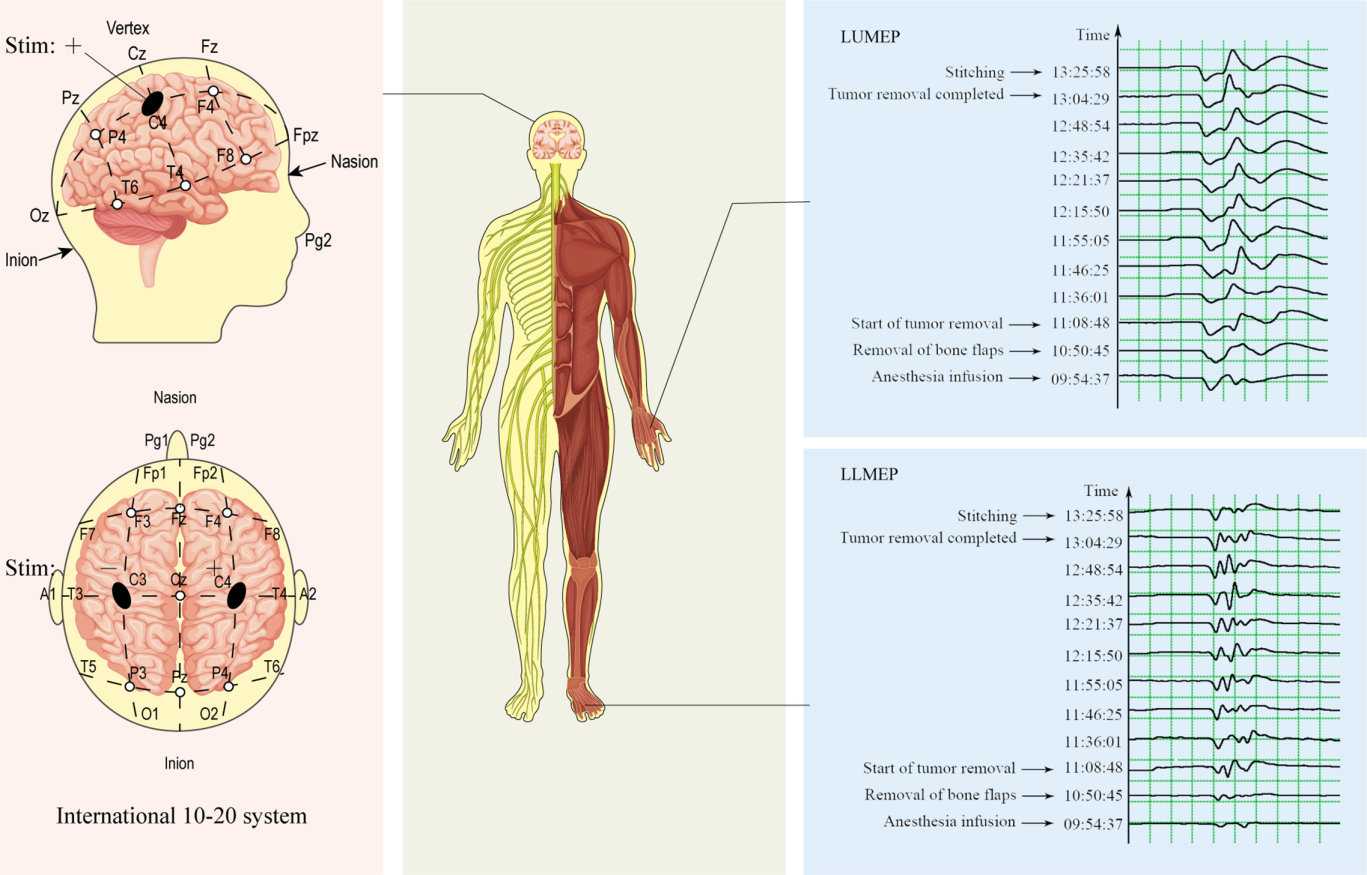
Diagram of intraoperative Tce-MEPs monitoring. During intraoperative monitoring of the Tce-MEPs amplitude on the left side of the patient, the polarity was switched by software, and positive and negative pulses were delivered through electrodes placed at C4 and C3, respectively. The amplitudes of the left upper Tce-MEPs (LUMEPs) and left lower Tce-MEPs (LLMEPs) were recorded by active electrodes placed over the belly of the contralateral thenar and abductor hallucis muscles.

Transcranial electrical MEPs and SSEPs were set up using standard half-inch subcutaneous needles at all stimulation and recording sites. Monitoring was performed by a trained neurophysiology technician or neurophysiologist. For Tce-MEPs stimulation, anodal pulses were delivered through electrodes placed at C3 and C4, and polarity switching was accomplished via software control (Figure 3). Tce-MEPs were recorded with an active electrode placed over the belly of the contralateral thenar and abductor hallucis muscles with a reference over the tendon of the muscle. Moreover, in the monitoring of SSEPs, tibial and median nerve SSEPs were stimulated at standard locations on the ankle and wrist, respectively. SSEPs were recorded with active electrodes at the lateral scalp (C3′ and C4′ are 2 cm posterior to the C3 and C4 locations of the international 10–20 system) for the median nerve and at the midline scalp (Cz') for the tibial nerve. These active leads were referred to as Fz.

An Xltek Protektor32 monitoring system (DBA Excel-Tech Ltd., Oakville, Canada) was used for the stimulation and recording. For the SSEPs and MEPs, the filter settings were 30–1,000 Hz. The analysis time was 50 milliseconds for median nerve stimulation and 100 milliseconds for tibial stimulation. SSEPs were the averages of 300 sweeps, and Tce-MEPs were the responses to a single train of stimulation. The tibial and median nerves were stimulated using a 0.2-millisecond duration pulse at intensities of 50 and 25 mAmps, respectively. Tce-MEPs were stimulated using a train of 4–8 pulses with an interstimulus interval of 2 milliseconds and an intensity between 150 and 400 V. SSEPs were monitored at computer-controlled 10-minute time intervals throughout the procedure. Tce-MEPs were manually monitored at 5- to 15-min intervals and were occasionally delayed to eliminate patient movement during critical portions of the surgical procedure. The same intensity of stimulation was maintained throughout the procedure for each patient.

The evoked potential (EP) information was first collected after 40 min of anesthesia to allow for the muscle relaxants used during anesthesia induction to be metabolized. Baseline EP measurements were taken before dexmedetomidine administration and consisted of 2–4 measurements. Dexmedetomidine was used at a continuous infusion of 0.5 μg/kg/h and was stopped after a 1-h infusion. We collected the EP information when the dexmedetomidine was stopped.

The data collected included the baseline patient characteristics (age, sex, height, weight); the intraoperative vital signs, which were collected and recorded by the study coordinator at baseline (T0) and at the end of dexmedetomidine administration (T1); and the intraoperative vital signs included the ASA classification, systolic blood pressure (SBP), diastolic blood pressure (DBP), mean arterial pressure (MAP), heart rate (HR), and temperature (T) (Figure 2). To evaluate the drug effects, the following parameters were used: for the median nerve SSEPs, the N20 latency (maximum negative peak) and N20-P22 amplitude (maximum negative peak-maximum positive peak) were recorded; for the tibial nerve SSEPs, the P37 latency (maximum positive peak) and P37-N45 amplitude (maximum positive peak-maximum negative peak) were recorded; and for the Tce-MEPs, the maximum peak-to-peak of the abductor hallucia amplitude was recorded. If data were missing, they were neither imputed nor included in the statistical analysis.

### Statistical analysis

We collected IONM data from patients during craniotomy with propofol-sevoflurane combined anesthesia at baseline (T0) and at the end of the 1-h dexmedetomidine infusion (T1). Subsequently, we calculated the percentage decrease in EPs and compared the differences among the dexmedetomidine group, the negative control group, the positive control group, and the fluctuating group. A *t*-test or nonparametric test was used to compare the differences in the percentage decrease in EPs and the absolute values of the differences in the percentage decrease in bilateral Tce-MEPs in the four groups according to whether the samples in each group conformed to a normal distribution.

**Figure 4:**
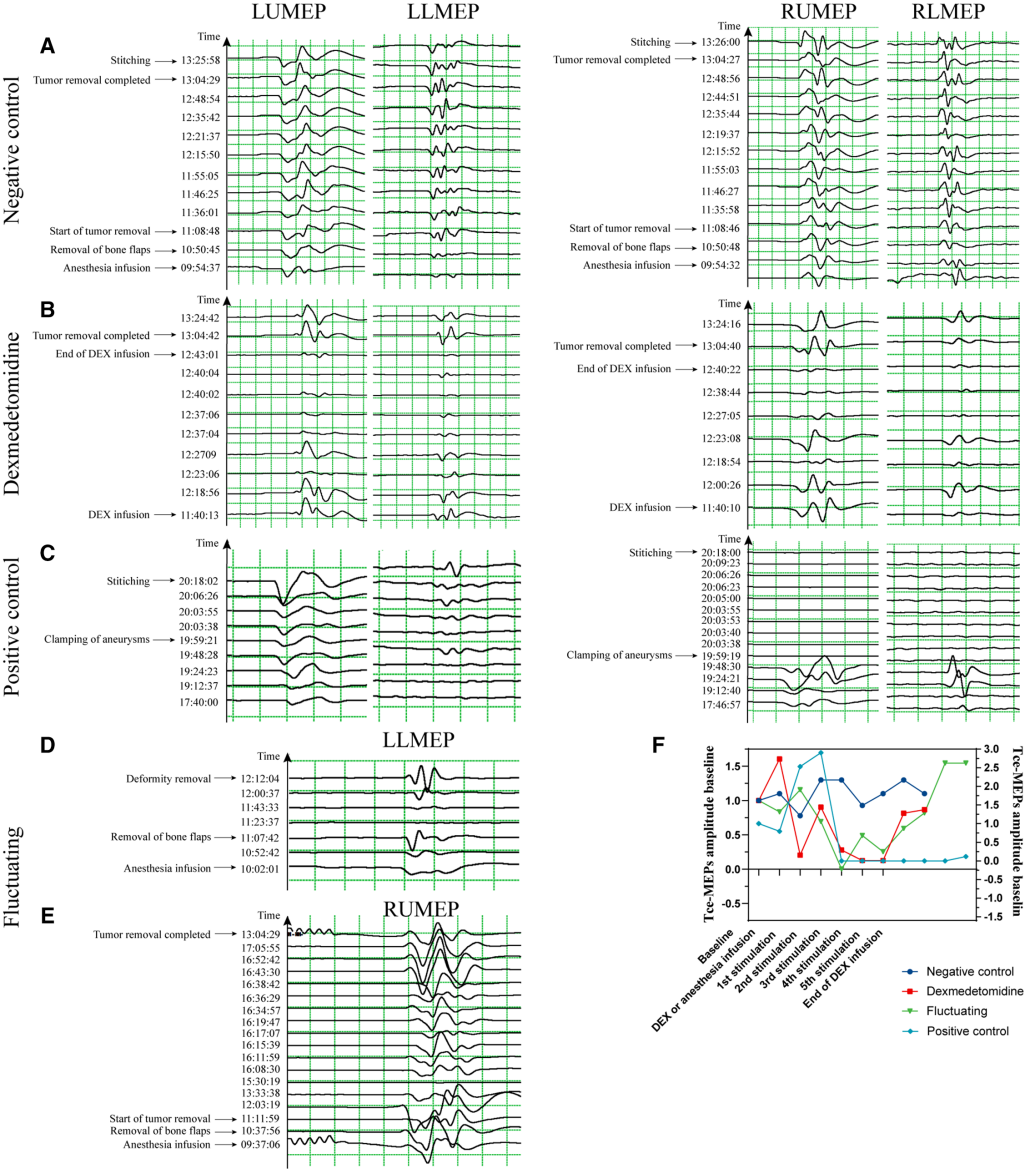
Typical images of Tce-MEPs amplitudes over time in the 4 groups. (**A**) is an image of a 57-year-old woman with a right temporal lobe glioblastoma undergoing resection of a brain lesion with a −30%∼27% decrease in the percentage of the Tce-MEPs amplitude, and the patient's pre- and postoperative muscle strength assessments were both grade V. (**B**) is an image of a 51-year-old woman with a diffuse astrocytoma undergoing occipital lobe lesion excision with a 30%∼86% decrease in the percentage of the Tce-MEPs amplitude; the patient suffered no motor deficits after surgery. It is clear that the decrease in the Tce-MEPs amplitude occurred approximately 37 min after the infusion of dexmedetomidine, and the Tce-MEPs amplitude continued to decrease until 20 min after the dexmedetomidine infusion was stopped. (**C**) is an image of a 47-year-old woman with an intracranial aneurysm who underwent intracranial aneurysm clamping. The percentage of the Tce-MEPs amplitude decreased by 88%∼100% on the right side, while there was no decrease on the left side. The patient developed right hemiparesis after the operation. (**D**) and (**E**) are images of a 52-year-old woman with a cerebrovascular malformation undergoing resection of a skull base lesion and a 56-year-old man with a cavernous sinus-occupying lesion undergoing resection of a cavernous sinus lesion, respectively. Neither of the two patients had motor deficits postoperatively, although a decrease in the Tce-MEPs amplitude was observed in both patients. (**F**). Time course of RUMEP amplitude variability in the four groups.

Considering that both dexmedetomidine addition and discontinuation require a certain amount of time for metabolism, which was reflected in the IONM data with a certain degree of delay (Figure 4), after communicating with the anesthesiologists, the percentage decrease in EPs in the dexmedetomidine group was defined as follows: (the maximum EPs after dexmedetomidine was added—the minimum EPs after dexmedetomidine was discontinued)/the maximum EPs after dexmedetomidine was added * 100%. The percentage decrease in EPs in the other 3 groups was calculated as follows: (EPs when dexmedetomidine was added—EPs when dexmedetomidine was discontinued)/EPs when dexmedetomidine was added * 100%. The absolute values of the differences in the percentage decrease in the bilateral Tce-MEPs were defined as the absolute value of the difference between the proportion of decline in the left Tce-MEPs and the proportion of decline in the right Tce-MEPs.

Spearman's rank correlation analysis was used to assess the correlation between the percentage decrease in Tce-MEPs and the grade of postoperative muscle strength and the decrease in muscle strength in these four groups. A decrease in muscle strength was defined as the difference between the preoperative muscle strength classification and the postoperative muscle strength classification. Unless otherwise stated, the data are reported as the mean ± SD (95% confidence interval) or *n* (%). All P values are bilateral, and differences were considered statistically significant at *α* = 0.05. All analyses were performed using SPSS 23.0 statistical software.

## Results

### Demographic and clinical characteristics

In this study, we reviewed the neurophysiological monitoring data and postoperative motor strength scores of 47 patients (22 females (46.81%) and 25 males (53.19%) with a mean age of 49 years) who underwent craniotomy under propofol-sevoflurane combined anesthesia, of whom 10 patients were additionally treated with dexmedetomidine. Table 1 summarizes the demographic and clinical characteristics of the patients in the four groups, including their age, sex, weight, height, ASA classification, comorbidities, surgical position, and pre- and postoperative muscle strength. There were no significant differences among the 4 groups.

**Table 1:** Patient demographic and clinical characteristics.

	Negative control (*n* = 13)	Dexmedetomidine (*n* = 10)	Positive control (*n* = 11)	Fluctuating (*n* = 13)
Age (y)	46.0 ± 18.4	51.9 ± 14.3	48.1 ± 12.9	51.7 ± 16.1
Male sex, *n* (%)	6 (46.2)	6 (60)	7 (63.6)	6 (46.2)
Weight (kg)	63.2 ± 13.8	60.5 ± 12.5	64.7 ± 12.2	63.5 ± 18.1
Height (cm)	162.5 ± 4.9	161.8 ± 7.4	163.5 ± 6.4	160.5 ± 19.8
ASA classification, *n* (%)				
Ⅰ	0 (0)	0 (0)	0 (0)	0 (0)
Ⅱ	10 (76,9)	8 (80)	8 (72.7)	9 (69.2)
Ⅲ	3 (23.1)	2 (20)	3 (27.3)	4 (30.8)
Disease, *n* (%)				
Tumor	6 (46.2)	9 (90)	8 (72.7)	6 (46.2)
Aneurysm	4 (30.7)	1 (10)	2 (18.2)	4 (30.7)
Other	3 (23.1)	1 (10)	1 (9.1)	3 (23.1)
Comorbidities, *n* (%)				
Any	4 (30.8)	4 (40)	5 (45.5)	9 (69.2)
Hypertension	3 (23.1)	4 (40)	4 (36.4)	6 (46.2)
Diabetes mellitus	1 (7.7)	0 (0)	0 (0)	3 (23.1)
COPD	0 (0)	0 (0)	0 (0)	0 (0)
Asthma	0 (0)	0 (0)	0 (0)	0 (0)
Other	1 (7.7)	0 (0)	1 (9.1)	4 (30.8)
Surgery, *n* (%)				
Resection intracranial space-occupying lesion	8 (61.5)	9 (90)	9 (81.8)	8 (61.5)
Intracranial aneurysmal clipped operation	5 (38.5)	0 (0)	2 (18.2)	4 (30.8)
Other	0 (0)	1 (10)	0 (0)	1 (7.7)
Surgical positioning				
Supine	13 (100)	8 (80)	10 (90.9)	10 (76.9)
Prone	0 (0)	1 (10)	0 (0)	0 (0)
Lateral	0 (0)	1 (10)	1 (9.1)	3 (23.1)
Preoperative muscle strength (grade)	4.9 ± 0.3	4.9 ± 0.3	4.8 ± 0.4	4.9 ± 0.3
Postoperative muscle strength (grade)	5 ± 0	5 ± 0	3 ± 1.6	4.7 ± 0.5

### Dexmedetomidine-induced decreases in the Tce-MEPs amplitude are similar to those induced by surgery

**Figure 5:**
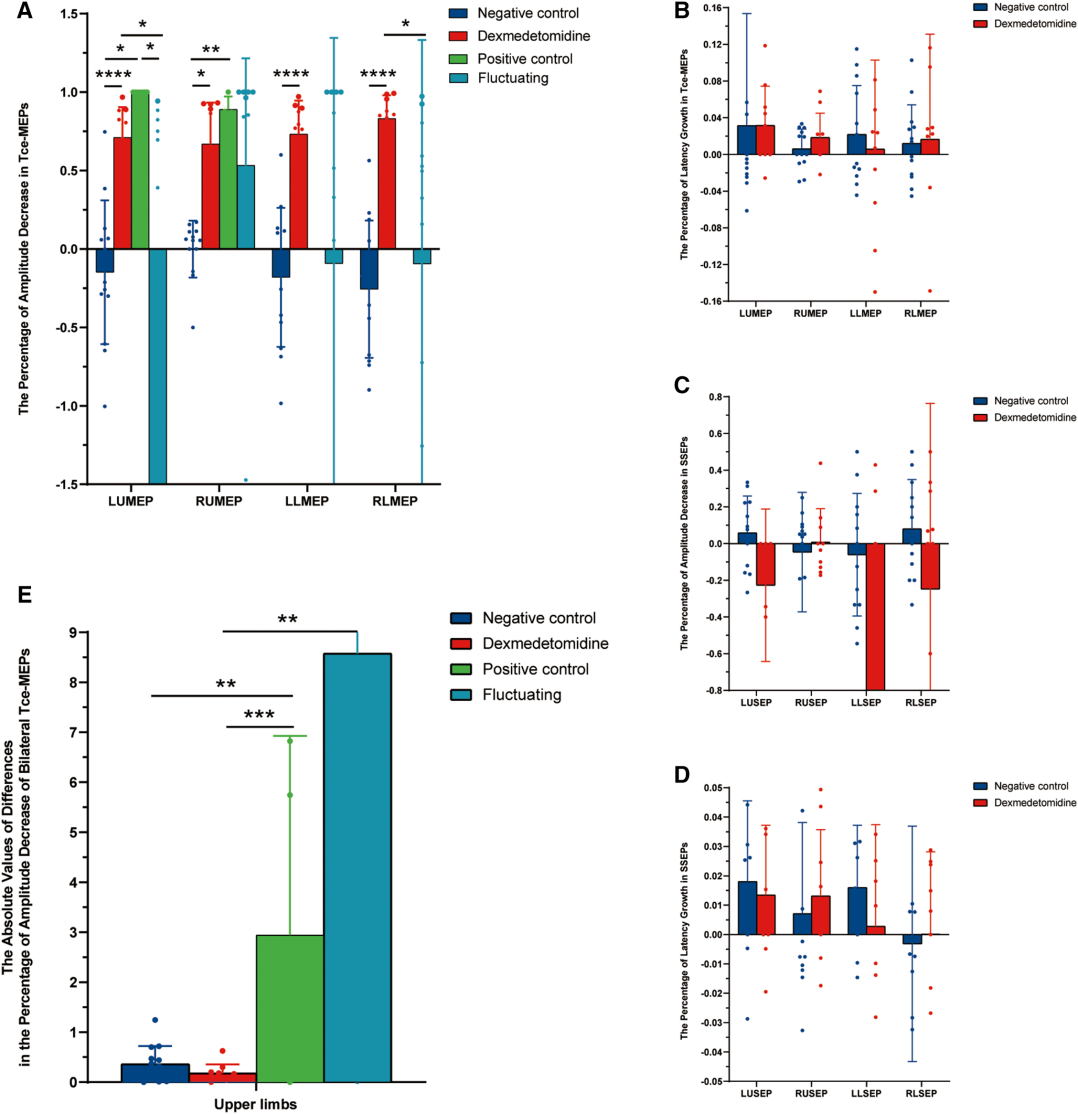
Histograms of the intraoperative neurophysiological monitoring parameters in the four groups. (**A**) The percentage of amplitude decrease in Tce-MEPs. Dexmedetomidine *vs.* negative control: LUMEP 0.71 ± 0.19 vs*.* −0.15 ± 0.46, *P* < 0.0001; RUMEP 0.67 ± 0.26 vs*.* −0.001 ± 0.18, *P* < 0.0001; RLMEP 0.83 ± 0.15 vs*.* −0.26 ± 0.44, *P* = 0.012; LLMEP 0.73 ± 0.21 vs*.* −0.18 ± 0.44, *P* < 0.0001. (**B**) The percentage of latency growth in Tce-MEPs. No statistically significant difference was found. (**C**) The percentage of amplitude decrease in SSEPs. No statistically significant difference was found. (**D**) The percentage of latency growth in SSEPs. No statistically significant difference was found. (**E**) The absolute value of differences in the percentage of amplitude decreases in bilateral Tce-MEPs. Dexmedetomidine *vs.* positive control: 0.18 ± 0.17 vs*.* 2.9 ± 3.8, *P* = 0.001; Dexmedetomidine vs*.* fluctuating group: 0.18 ± 0.17 vs*.* 8.57 ± 17.4, *P* = 0.003; Negative control vs*.* positive control: 0.36 ± 0.35 vs*.* 2.9 ± 3.8, *P* = 0.009.

We collected data on Tce-MEPs at baseline (T0) and at the end of the 1-h dexmedetomidine infusion (T1). Changes in the amplitude and latency of the Tce-MEPs were analyzed postoperatively. The data from the four groups were compared pairwise, and the results are presented in Figure 5. We found that the Tce-MEPs amplitude significantly decreased (*p* < 0.05) in the dexmedetomidine group compared with the negative control group, suggesting that the application of dexmedetomidine as an adjunct to TIVA during craniotomy resulted in a decrease in the Tce-MEPs amplitude. Furthermore, there was no significant difference in the decrease in the Tce-MEPs amplitude between the dexmedetomidine group and the positive control group or between the dexmedetomidine group and the fluctuating group, indicating that the application of dexmedetomidine might mask the warning of a neurological deficit during intraoperative monitoring and increase the risk of misjudgment (Figure 5A).

We next sought to analyze whether the amplitudes of SSEPs among the 4 groups were able to distinguish the dexmedetomidine group from the other 3 groups. However, the SSEPs were relatively stable and not significantly different between the dexmedetomidine and control groups (Figure 5C). In addition, the Tce-MEPs and SSEP latency were also calculated and the percentage of latency increase was statistically analyzed; however, there were no statistical differences among the four groups (Figures 5B,D).

### Surgery-related decreases in Tce-MEPs lead to motor deficits

The postoperative motor strength of the 4 groups is shown in Table 1. Motor deficits were used to indirectly demonstrate that intraoperative brain motor function areas were damaged. The majority of the limbs in the positive control and fluctuating groups that showed a decrease in the Tce-MEPs amplitude developed motor deficits after surgery. The correlations between the percentage of decrease in the Tce-MEPs amplitude and the postoperative muscle strength scale values and the decrease in muscle strength in the two groups is shown in Table 2. In the positive control group, Spearman rank correlation analysis revealed a moderate negative correlation between the percentage decrease in the Tce-MEPs amplitude and patients' postoperative muscle strength (*p* < 0.05) and a moderate positive correlation between the percentage decrease in the Tce-MEPs amplitude and the decrease in muscle strength (*p* < 0.05). In contrast, there was no correlation between the percentage decrease in the Tce-MEPs amplitude and the grade of postoperative muscle strength and decrease in muscle strength in the fluctuating group (*p* > 0.05). In addition, there was no significant decrease in postoperative motor function in either the dexmedetomidine group or the negative control group, indicating that the intraoperative use of dexmedetomidine did not affect the prognosis of the patients.

**Table 2:** Spearman rank correlation between the percentage decrease in the Tce-MEPs amplitude and the grade of postoperative muscle strength and the decrease in muscle strength.

Percentage decrease in the Tce-MEPs amplitude	Positive group (*n* = 11)		Fluctuating group (*n* = 13)	
	*r*	*P-*value	*r*	*P-*value
Postoperative muscle strength (grade)	0.507	0.016	0.075	0.715
Decline of muscle strength (grade)	−0.629	0.002	−0.056	0.788

### The effects of dexmedetomidine on Tce-MEPs are bilateral

To investigate the effects of dexmedetomidine and actual brain injury on the decrease in the Tce-MEPs amplitude, we compared the absolute values of the difference in the percentage decrease in the Tce-MEPs amplitude bilaterally among the four groups (Figure 5). Because the unilateral or bilateral lower limb Tce-MEPs amplitudes were not measured in five patients in the positive control group, only six patients for whom the absolute value of the percentage difference in the lower limb Tce-MEPs amplitude decreased in the positive control group remained after subtracting the data from the left and right sides, which was too small to be analyzed. Therefore, only the upper limb data were compared and analyzed in this study (Figure 5E).

In the negative control group, TIVA drugs simultaneously affected the IONM parameters; thus, the amplitude fluctuation trends of the Tce-MEPs on the left and right sides of patients were similar, and the absolute values of the differences in the percentage decrease in the bilateral Tce-MEPs amplitudes were small. In the positive control group, the Tce-MEPs amplitude decreased mainly on the side of the brain injury, which was often unilateral, resulting in differences in the percentage decrease of the bilateral Tce-MEPs.

The absolute values of the differences in the percentage decrease in bilateral Tce-MEPs were significantly different between the negative and positive control groups (*p* = 0.009). There was no statistically significant difference between the dexmedetomidine group and the negative control group or between the negative control group and the positive control group (*p* = 0.001). This indicated that, similar to the negative control group, the percentage decrease in the amplitude of Tce-MEPs in both upper extremities was similar in the dexmedetomidine group, and we therefore concluded that the percentage decrease in the amplitude of Tce-MEPs in both upper extremities was similar in the two groups. Therefore, the effects of dexmedetomidine and TIVA anesthetic drugs on intraoperative Tce-MEPs amplitudes were bilateral for both drugs.

## Discussion

Dexmedetomidine is a highly selective *α*2-adrenergic agonist. Unlike propofol, dexmedetomidine has less inhibitory effects on cortical and thalamocortical activity, and while sedated, the patient remains awake and is easily aroused during neurosurgical procedures (29). However, it has been reported that the addition of dexmedetomidine during central nervous system surgery could affect the accuracy of EPs. In the present study, IONM data from 47 craniotomy patients treated with dexmedetomidine addition regimen and conventional propofol-sevoflurane combined anesthesia regimen were retrospectively analyzed. We found that dexmedetomidine had an inhibitory effect on the Tce-MEPs amplitude during craniotomy, which is consistent with previous studies (26–28). The decreases resulting from the use of dexmedetomidine were similar to those resulting from surgery, and the use of dexmedetomidine masked the warning of a neurological deficit on intraoperative monitoring. Moreover, we demonstrated that the inhibitory effect of dexmedetomidine on Tce-MEPs is often bilateral, which will provide a new way to determine Tce-MEPs amplitude decreases caused by the side effects of dexmedetomidine.

The effects of dexmedetomidine on neuroelectrophysiology were first studied in spinal cord surgery. Mahmoud et al. (26, 30) reported 1 case study and 1 experimental study on adolescents with scoliosis deformity who underwent spinal surgery. Dexmedetomidine, an anesthetic adjuvant for TIVA, significantly attenuated the amplitude of Tce-MEPs at plasma target concentrations of both 0.6∼0.8 ng/ml and 0.5 μg/kg/h. A dose‒response relationship was found between the dose of dexmedetomidine and the reduction or decrease in the amplitude of Tce-MEPs. Liu et al. (31) conducted a randomized, double-blinded, placebo-controlled study and reported that TIVA combined with a loading dose of dexmedetomidine (1 μg/kg over 10 min) followed by a constant-rate infusion (0.5 μg/kg/h) had an inhibitory effect on IONM parameters, including significant decreases in the SSEP amplitude and MEP amplitude as well as an increase in the SSEP latency, although the IONM parameters did not significantly differ between the group receiving dexmedetomidine at a constant infusion rate (0.5 μg/kg/h) and the control group. Moreover, the authors recommended that IONM be recorded at intervals of less than 15 min during the first 30 min after dexmedetomidine (1 μg/kg) injection to avoid missing statistically significant changes in the amplitude and latency of evoked potentials. However, several other studies in spinal surgery have reported no statistically significant difference in IONM data between the dexmedetomidine-added regimen and the conventional propofol-sevoflurane combination anesthesia regimen in either adults or children (13, 24, 25). Therefore, whether dexmedetomidine is safe and recommended for application as an option for adjunctive medication to TIVA in spinal surgery remains inconclusive, and further experimental and clinical studies are needed.

There is even less clinical evidence on the safety and feasibility of dexmedetomidine as an adjuvant to TIVA in craniotomy. Our study demonstrated that SSEPs are not affected by dexmedetomidine, which is consistent with the results of previous experimental and clinical studies. Lee et al. (27) reported that continuous infusion of 0.5 μg/kg/h dexmedetomidine had a significant inhibitory effect on the amplitude of Tce-MEPs during IONM in brain tumor surgery. However, in contrast to our findings, Pacreu et al. (20), by prospectively studying 40 patients, demonstrated that 0.5 μg/kg/h dexmedetomidine had no effect on IONM parameters in patients who underwent brainstem or supratentorial craniosurgery and could reduce the need for propofol. We believe that the following four reasons may explain this difference. First, the anesthesia regimens used in the dexmedetomidine group differed. Second, Pacreu et al. only recorded Tce-MEPs and SSEPs at baseline, 15, 30, 45 min, and at the end of surgery, while we recorded from start to finish. Statistically significant changes in Tce-MEPs were difficult to observe within the first 30 min after dexmedetomidine infusion in our study. Third, both studies were small sample size clinical studies with many differences in the surgical methods, monitoring equipment, patient ethnicity, etc., and the findings were idiosyncratic and less representative. Therefore, as mentioned earlier, studies of dexmedetomidine in cranial surgery are limited, and it is still controversial whether dexmedetomidine inhibits the Tce-MEPs amplitude, so future multicenter collaborative studies are highly needed.

Moreover, in the present study, the Tce-MEPs amplitude did not decrease immediately after dexmedetomidine infusion but fluctuated down after a period of time after dexmedetomidine infusion; similarly, the Tce-MEPs amplitude increased after dexmedetomidine was discontinued for a period of time. We hypothesized that this delayed phenomenon may be related to the differences in the distribution and clearance of dexmedetomidine in patients (32, 33). Pharmacokinetic studies have shown that body size and hepatic function significantly influence the pharmacokinetic profile of dexmedetomidine (29).

It is well known that both intraoperative application of dexmedetomidine and medical brain injury can lead to a decrease in the amplitude of Tce-MEPs. Therefore, identifying a method capable of differentiating the cause of the decrease in the Tce-MEPs amplitude is another strength of the present study. By comparing the absolute values of the differences in the percentage decrease in the bilateral Tce-MEPs of the four groups, the positive control group showed a decrease in the Tce-MEPs amplitude only on the brain injury side, which was often unilateral, whereas the effects of dexmedetomidine and TIVA drugs on the Tce-MEPs amplitude were bilateral. By comparing the 4 groups, we initially identified the typical characteristics of the dexmedetomidine group: craniotomy patients treated with dexmedetomidine experienced a bilateral decrease in the Tce-MEPs amplitude but did not suffer postoperative motor deficits. This finding is important for neurosurgeons to determine the cause of the Tce-MEPs amplitude decrease more clearly and adjust the surgical plan in time, which is helpful for reducing the risk of iatrogenic injuries and improving the prognosis of motor function in patients undergoing craniotomy.

There are still some limitations in the present study. First, this was a retrospective clinical study. The sample size was small due to time constraints and missing data. Only 10 patients received dexmedetomidine in addition to their anesthesia regimen. However, this study revealed that dexmedetomidine has an inhibitory effect on IONM, and neurosurgeons can identify this effect by comparing the absolute values of the differences in the percentage decrease in the bilateral Tce-MEPs amplitudes. There is no conclusive evidence regarding the safety and feasibility of using dexmedetomidine as an adjunct to TIVA in craniotomy, and further research and studies are needed. Second, this study did not collect patients' immediate postoperative muscle strength scores and was unable to analyze patients' postoperative motor function recovery on a continuous basis. Our future work will focus on collecting data from larger clinical cohorts to comprehensively evaluate the prospects of the practical application of dexmedetomidine in craniotomy.

## Conclusion

In summary, although dexmedetomidine does not affect the prognosis of patients undergoing craniotomy, the side effects of dexmedetomidine may seriously interfere with the interpretation of IONM results and therefore jeopardize the reliability of intraoperative MEP monitoring. When the Tce-MEPs amplitude decreases, it is difficult for the neurosurgeon to determine whether this decrease is due to iatrogenic lesions or the side effects of dexmedetomidine. The potential risks and benefits of adding dexmedetomidine should be carefully evaluated before using it as an adjunct to TIVA for craniotomy. If dexmedetomidine is used, the cause of the decrease in the Tce-MEPs amplitude can be indirectly determined by whether the decrease is bilateral. If there is a unilateral decrease in the Tce-MEPs amplitude, one should be alerted to functional brain injury; if there is a bilateral decrease in the Tce-MEPs amplitude, dexmedetomidine may be the influence or both functional brain areas may be injured, and further communication with the anesthesiologist is needed to reduce the use of dexmedetomidine to further determine the cause of the decrease in the Tce-MEPs amplitude.

## Data Availability

The original contributions presented in the study are included in the article/Supplementary Material, further inquiries can be directed to the corresponding authors.
