# Corrigendum: Effect of dexmedetomidine on somatosensory- and motor evoked potentials in patients receiving craniotomy under propofol-sevoflurane combined anesthesia

**DOI:** 10.3389/fsurg.2024.1480011

**Published:** 2024-08-28

**Authors:** Xue Yang, Xinyi Zhang, Puxuan Lin, Zeheng Liu, Shuhang Deng, Shanwen Liang, Xinyi Zhu, Qianqian Qiao, Qianxue Chen

**Affiliations:** ^1^ Department of Neurosurgery, Renmin Hospital of Wuhan University, Wuhan, China; ^2^ Department of Anesthesiology, Renmin Hospital of Wuhan University, Wuhan, China

**Keywords:** dexmedetomidine, transcranial motor-evoked potentials, intraoperative neurophysiological monitoring, neurosurgery, prognosis

A Corrigendum on Effect of dexmedetomidine on somatosensory- and motor-evoked potentials in patients receiving craniotomy under propofol-sevoflurane combined anesthesia By Yang X, Zhang X, Lin P, Liu Z, Deng S, Liang S, Zhu X, Qiao Q, Chen Q (2024). Front. Surg. 11:1386049. doi: 10.3389/fsurg.2024.1386049

**Incorrect Affiliation**

In the published article, there was an error in affiliations [^1^Department of Neurosurgery, Wuhan University Renmin Hospital, Wuhan, China, ^2^Department of Anesthesiology, Wuhan University Renmin Hospital, Wuhan, China]. Instead of “[^1^Department of Neurosurgery, Wuhan University Renmin Hospital, Wuhan, China, ^2^Department of Anesthesiology, Wuhan University Renmin Hospital, Wuhan, China]”, it should be “[^1^Department of Neurosurgery, Renmin Hospital of Wuhan University, Wuhan, China, ^2^Department of Anesthesiology, Renmin Hospital of Wuhan University, Wuhan, China]”.

The authors apologize for this error and state that this does not change the scientific conclusions of the article in any way. The original article has been updated.

